# Organizational readiness for implementing clinical decision support tools in chronic pain care: a mixed methods evaluation

**DOI:** 10.3389/frhs.2025.1613208

**Published:** 2025-08-05

**Authors:** Miranda J. Reid, Jyotsna Gutta, Katy Ellis Hilts, Magda Knight, Julie DiIulio, Lori Bilello, Mario El Hayek, Khoa A. Nguyen, Christopher A. Harle, Ramzi G. Salloum

**Affiliations:** ^1^ Health Outcomes and Biomedical Informatics, University of Florida, Gainesville, FL, United States; ^2^ Health Policy and Management, Indiana University, Indianapolis, IN, United States; ^3^ Applied Decision Science, Cincinnati, OH, United States

**Keywords:** organizational readiness, implementation science, clinical decision support, mixed methods, chronic pain

## Abstract

**Introduction:**

Clinical decision support (CDS) tools have the potential to enhance treatment outcomes in chronic pain care, yet their successful implementation depends on the readiness of both providers and clinical staff. The purpose of this study was to assess the readiness for a CDS tool and identify implementation strategies.

**Methods:**

A mixed methods approach combined Organizational Readiness for Implementing Change (ORIC) surveys (*n* = 24 providers; *n* = 31 clinical staff) and semi-structured interviews (*n* = 7 providers; *n* = 3 clinical staff). Quantitative data were analyzed using descriptive statistics and inferential tests, while qualitative data were coded using a rapid qualitative analysis approach.

**Results:**

Both the ORIC surveys and semi-structured interviews revealed high perceived organizational readiness for implementation across clinics and roles. There was variation in change efficacy, commitment to change, and overall readiness by clinics (*p* > 0.05) and between roles (*p* < 0.01), with providers demonstrating statistically significant lower ORIC scores than clinical staff. The qualitative results added nuance, with participants identifying patient and provider level barriers to implementation (e.g., technological literacy, low relative priority). However, these barriers were perceived as surmountable with implementation strategies (e.g., technological support, training and materials for providers and staff).

**Discussion:**

The study highlights the benefits of a mixed methods approach to assessing the readiness. Variation in ORIC scores can help target support resources to clinics and roles with lower perceived readiness. Interviews provide an essential opportunity to identify local barriers and acceptable implementation strategies to build stakeholder support. Combined, these approaches allow for a holistic approach to inform tailored implementation support.

## Introduction

Chronic non-cancer pain continues to be a major public health concern, affecting an estimated 1 in 5 Americans each year (1). Primary care providers often face time constraints which make it difficult to efficiently assess patients’ pain and function, understand patient goals, and recommend appropriate pain treatments (2). Clinical decision support (CDS) tools in electronic health records (EHRs) may help collect and present this information thus overcoming provider-level barriers to shared decision-making with patients regarding the best options to address their pain (3). However, while these technologies are promising, implementation of health IT often fails in these settings (4, 5). It is important to understand organizational readiness and how to mitigate potential barriers to ensure new health IT is useable and aligned with the organizational structures, clinical workflows, user information needs, and decision-making practices (6–8). Further, understanding differing aspects of readiness in an organization can help in determining the capacity and willingness to implement evidence-based interventions (9, 10).

Weiner (2009) defines organizational readiness as a multi-component construct that encompasses organization members’ shared resolve (change commitment) to implement an innovation and their belief in their combined capacity to do so (change efficacy) (9). Although it is common to have high perceived organizational readiness in the pre-implementation phase across many clinical settings (11–13), Organizational Readiness for Implementing Change (ORIC) scores can still prove useful by identifying groups that are relatively less prepared. Groups with lower baseline ORIC scores have the potential for the greatest improvement in intervention delivery (14). While quantitative measures of readiness such as ORIC provide valuable insights into an organization's baseline readiness, they often lack the nuance needed to capture more complex dynamics within healthcare organizations.

Qualitative measures, on the other hand, allow for a deeper exploration of organizational culture, interpersonal relationships, and hidden concerns that may not be easily captured by quantitative instruments alone. They allow participants to prioritize barriers and offer feedback on proposed strategies. As such, mixed-methods approaches can be used to identify additional barriers and relevant strategies, even in settings with high baseline ORIC scores (11).

The purpose of this study was to identify contextual factors influencing the implementation of CDS tools for chronic non-cancer pain care among primary care providers and clinical staff to inform the development of tailored implementation strategies. We employed a formative evaluation using a quantitative survey and qualitative interviews to assess readiness and potential CDS implementation barriers among provider and clinical staff at these clinics. This formative evaluation is part of a research project to adapt and optimize the implementation of two previously implemented interoperable CDS tools, My Pain and PainManager (15, 16), within primary care clinics in the University of Florida (UF) Health system. Findings from our quantitative and qualitative analyses provided complementary information to inform the development of strategies to address barriers and to support clinic personnel in implementing these tools.

## Methods

Using a concurrent parallel design, we conducted a mixed methods analysis of survey and semi-structured interviews on organizational readiness to implement MyPAIN and PainManager. MyPAIN is a patient-facing web application that leverages Fast Healthcare Interoperability Resources (FHIR) standards to collect patient-reported pain information. This tool enables patients to provide detailed insights into their pain experiences, functional limitations, and treatment preferences, empowering them to actively participate in their care. PainManager is a clinician-facing web application that interfaces with the EHR using FHIR standards. PainManager synthesizes patient-reported data from MyPAIN with clinical information from the EHR, showing providers a comprehensive view of their patients’ pain conditions.

Eight adult primary care clinics were selected due to the relatively high volume of chronic non-cancer pain patients seen in these clinics compared to other UF Health clinics in the Jacksonville, FL area. Eligible participants included primary care providers (e.g., physicians, nurse practitioners, advanced practice providers) and clinical staff (e.g., medical assistants, registered nurses, office managers) who care for patients with chronic non-cancer pain in participating clinics. Data were collected from July 2023 through November 2023 as formative research prior to implementing the CDS tool.

To quantitatively assess readiness, the ORIC survey was administered to providers and staff at the participating clinics. ORIC is a validated survey that uses a 12-item scale to measure respondents’ commitment to change and change efficacy. Individual items assess respondent agreement with statements using a 5-point Likert scale, with responses ranging from “Disagree” to “Agree”. In order to briefly and reliably assess these concepts, statements are broad and focus on “organizational members’ shared resolve to implement a change.” (10) The survey was administered, and data were managed using REDCap (version 14.7.2). To enhance response rates, respondents were offered a $10 gift card incentive upon completion survey completion. Research team staff visited the selected clinics to facilitate the survey administration process. Informed consent was obtained from all participants prior to their participation in the study. Electronic data collected from the survey responses were stored securely per UF standards. This study received ethical approval from the University of Florida Institutional Review Board (IRB) prior to data collection (IRB202202793). All procedures were conducted in accordance with ethical guidelines, ensuring participant confidentiality, privacy, and voluntary participation.

Summary statistics (mean, standard deviation) were used to characterize responses to individual survey items and overall scores for change efficacy, change commitment, and organizational readiness. Two-sample t-tests assessed differences by role (provider vs. clinical staff), and ANOVAs were used to assess differences by clinic. Analyses were conducted using Stata (version 18.5).

Semi-structured interviews were conducted to qualitatively assess the readiness and elicit potential contextual factors influencing implementation of the CDS tools among clinic providers and staff. Seven medical directors representing the eight participating clinics were recruited to evaluate provider perspectives. Clinical staff (*n* = 3) were recruited from a subset of four clinics, purposively selected based on differences in clinic workflow and patient population. Interviewers wrote memos after each interview, and recruitment stopped after new themes were no longer emerging from subsequent interviews, indicating saturation was reached. Reaching saturation after ten interviews, likely results from their high “information power”, given the specificity of the sample and narrow aims of the interview guide (17). Participants were recruited over email and received a $20 gift card incentive for completing the interviews. The interview guide was designed by research staff with experience in human factors psychology and user-centered design to familiarize the interviewee with the CDS software, elicit barriers and facilitators, and explore tailored support strategies to address these barriers. In addition to open-ended questions on strategies, we assessed support strategies used in previous roll outs within the health system including trainings, educational materials, technical support, and clinical champions. To complement the broader assessment of organizational readiness in ORIC, the interviews highlighted specific barriers and strategies to address them. Interviews were conducted virtually, lasting between 30 and 45 minutes and were conducted and recorded with permission using Zoom (version 6.2.6).

Interview transcripts were analyzed using a rapid qualitative analysis approach (18, 19). To ensure reliability, data from each interview were extracted by 2 coders into a template based on the interview guide. Any conflicts were resolved by group discussion until consensus was reached (20). Data were transferred into two distinct respondent-by-domain matrices based on interviewee role (primary care providers, clinical staff) (18). Then two study staff members inductively coded the resulting matrices. After independently coding the matrices for four interviews, emerging themes were used to develop a codebook organized by Consolidated Framework for Implementation Research (CFIR) and Expert Recommendations for Implementing Change (ERIC) constructs (21–23). The CFIR was used to identify the contextual factors influencing implementation, whereas ERIC informed identification of implementation strategies to address identified barriers. The two study staff members met regularly to iteratively update the codebook and reconcile differences. All matrix data were coded by both study staff members, conflicts were resolved by group discussion with the larger team, and consensus was reached (20). Study staff involved in extraction and analysis kept memos continuously throughout the process to document decisions and emerging themes. Thematic saturation was reached when at least 70% of the participating sites reported the barrier or strategy.

## Results

### Survey results

A total of 55 surveys were completed across 8 pre-selected clinics, by providers (*n* = 24) and clinical staff (*n* = 31). Overall, respondents reported a high level of readiness for implementing a new tool for pain care into the existing EHR, with a mean ORIC score of 52.6 (out of 60). Both ORIC subdomains of change efficacy and commitment to change also had high scores, with mean scores of 30.7 and 21.9, respectively. Though both providers and clinical staff had high scores for readiness to implement a new tool, clinical staff were significantly more likely than providers (*p* < 0.01) to consider their clinic ready to implement a new tool in Epic® EHR (Table 1). Additionally, there were minor non-statistically significant variations in change efficacy, commitment to change, and total ORIC scores across clinics (Table 2). These variations were not associated with clinic patient volume, insurance status of patients, or urban vs. suburban setting.

**Table 1:** Provider to clinical staff variation in ORIC scores.

Role	Provider	Clinical staff	*p*-value
Change efficacy [mean (SD)]^a^	28.6 (1.0)	32.3 (0.8)	0.0055
Commitment to Change [mean (SD)]^b^	20.6 (0.8)	22.9 (0.6)	0.0190
Total Score [mean (SD)]^c^	49.2 (1.8)	55.2 (1.3)	0.0081

^a^ The minimum score for the Change Efficacy domain is 7, and the maximum score is 35.

^b^ The minimum score for the Change Commitment domain is 5, and the maximum score is 25.

^c^ The minimum total score for the ORIC is 12, and the maximum total score is 60.

**Table 2:** Variation in ORIC scores across clinics.

Clinic	A	B	C	D	E	F	G	H	*p*-value
Change efficacy [mean (SD)]^a^	33.5 (2.4)	28.9 (6.5)	31.7 (4.8)	29.3 (7.8)	30.0 (5.0)	31.2 (3.8)	31.6 (3.9)	29.4 (4.8)	0.16
Commitment to Change [mean (SD)]^b^	24.2 (1.2)	20.4 (4.9)	22.5 (3.2)	21.7 (5.5)	21.6 (3.5)	21.8 (3.5)	22.1 (3.9)	21.4 (2.6)	0.28
Total score [mean (SD)]^c^	57.7 (3.4)	49.3 (11.3)	54.2 (8.0)	51.0 (13.1)	51.6 (8.2)	53.0 (7.3)	53.8 (7.5)	50.8 (7.4)	0.21

^a^ The minimum score for the Change Efficacy domain is 7, and the maximum score for is 35.

^b^ The minimum score for the Change Commitment domain is 5, and the maximum score is 25.

^c^ The minimum total score for the ORIC is 12, and the maximum total score for is 60.

### Qualitative results

Ten participants (7 medical directors, 3 clinical staff) representing 8 clinics participated in interviews. Participants identified barriers at both the patient and provider levels and recommended corresponding implementation strategies (Table 3).

**Table 3:** CFIR barriers to readiness and ERIC strategies to support CDS implementation.

Medical directors and staff see these factors as impacting readiness for implementation	Strategies suggested by participants to resolve the problems
Patient needs (technological literacy)	Offering technical assistance
Absence of strategies to promote patient buy-in	Intervening with patients directly to promote uptake
Compatibility of the intervention with workflow	Capturing and sharing local knowledge
Low relative priority of the intervention	Providing training and educational materials

Participants emphasized the importance of balancing these two sets of concerns. For example, efforts to address patient technological needs (patient needs—CFIR construct) and improve their buy-in (patient buy-in—CFIR construct) could not come at the expense of compatibility with provider and staff workflow (workflow compatibility- CFIR construct), especially given the low relative priority of the intervention (relative priority- CFIR construct) compared to their existing tasks:

“I feel like you would have to piggyback on something that we're already doing. Like calling them. Like I said, we try to call them prior to, to remind them to come into their appointment. So, I guess the only way I can see that happen is if they call them and tell them during that reminder, oh, by the way, please remember to fill out your MyPain survey.” -Medical Director, Clinic G

Other participants suggested that the implementation team may need to promote MyPain to patients themselves (intervening directly- ERIC strategy) and offer technical assistance (tech assistance—ERIC strategy) to both patients and providers, instead of supporting clinics in intervening with patients, due to existing workflows and staffing limitations at their clinic (workflow compatibility—CFIR construct).

“I think the most important thing will be how to support the patients as our clinical staff is short. … As providers, … we're going to need more support at the beginning, but probably less and less as that goes on. But as we keep getting patients and patients, that is going to be … needed more on a recurrent basis. And it could be as simple as if the patient is referred—… later on receiving a call or a text saying, if you need help, please call us here.”—Medical Director, Clinic A

Overall, while many potential barriers were identified, participants had a high degree of confidence that they would be able to address them. This was true of primary care providers who believed they could leverage their strong existing relationships with their patients to overcome barriers:

“I think it'll be harder in certain patients who have managed their pain a certain way for a very long time and are resistant to change will have a difficult time using these. But I do think that you could get buy-in from almost anybody.” -Medical Director, Clinic C

And it was also true of clinical staff who were used to doing their best in an environment with significant staffing and workflow limitations:

“Well, short staff, [that’s] one. Everybody’s spread thin in a way, but nothing other than that. We get the job done, but it is tough days, but no real barriers other than that.”—Clinic Staff, Clinic E

Most clinics preferred a strong initial rollout, including training for staff and providers and educational materials (training and materials—ERIC strategy) with long-term technical support available on an as-needed basis (tech assistance—ERIC strategy). This held true across clinics with varying organizational readiness and across roles:

“So, I think the in-person training in a group setting, at least to get it rolled out with some physical flyer material. … But I think following that we'll more so probably lean on the phone and the email.”—Medical Director, Clinic C

“In the beginning, I would say everything [training, educational flyers] in the beginning to get us all where we need to be. And then afterwards, like I said, we can shoot out an email if we need extra help.”—Clinical Staff, Clinic B

After this initial support, participants suggested maintaining momentum by collecting and sharing stories of the barriers to implementation other clinics had faced and how they had overcome them (local knowledge- ERIC strategy). This could help promote compatibility with workflow and reduce effort for providers to get their questions answered.

“So people know that, okay, maybe I'm not the one going through this or maybe … addressing that barrier, that common problem or issue will answer their questions instead of them having to take the time to reach out.” -Medical Director, Clinic G

## Discussion

Overall, providers and clinical staff have a high perception of their organization's readiness to implement a CDS tool for chronic non-cancer pain care. The high change commitment, change efficacy, and total ORIC scores align with high perceived organizational readiness in the pre-implementation phase in many clinical settings (11–13). In some previous studies, ORIC scores were so high that they actually decreased from baseline with the development of an implementation support plan (12, 13). This finding may be because the process of identifying potential barriers, even if they are being addressed, may decrease perceptions of organizational readiness.

In our study, ORIC scores provided valuable insights into individual-level measures of change commitment and efficacy, revealing differences between providers and staff and between clinics. Although these differences were small, likely due to the limited sample size, they suggest that certain subgroups within the clinics may require more targeted support to achieve full readiness. Similarly, variation in readiness across clinics can inform the allocation of additional training and support resources. This tailored support is essential as without it, clinics with lower ORIC scores may experience fewer benefits from training and technical assistance (25). This result could compound existing differences in readiness leading to differential outcomes.

Qualitative data from the interviews added nuance to the survey results. While participants felt broadly ready, they were able to identify specific barriers to implementation, such as challenges with workflow integration, competing priorities, and the need for patient-facing support. Importantly, these barriers were perceived as surmountable, with participants suggesting strategies such as offering technical support and training to providers and staff, sharing success stories between clinics, and directly supporting patients. In previous research, having specific strategies for addressing barriers and confidence in overcoming them has aligned with higher ORIC scores (24). In this way, our qualitative findings corroborate the high perceived organizational readiness found in our survey results.

Insights gained from these interviews are crucial for developing tailored implementation support strategies that are responsive to the unique needs of each clinic. For example, while Clinic G expressed the ability to incorporate some patient support into their existing clinic workflow, Clinic A stated that all patient support would need to come from the implementation team. Interestingly, the ERIC strategies identified by participants were different than those that previous literature would have emphasized given the CFIR constructs identified. For example, to address the CFIR construct “relative priority”, the CFIR-ERIC matching tool would suggest conducting local needs assessments or consensus discussions, as well as directly working to increase demand or in fact mandating the change (22), while in our interviews, participants suggested that trainings and educational materials would raise awareness and thus raise the relative priority. These differences may indicate local knowledge about which approaches are most effective in each clinical setting.

This formative research is part of a larger study that is evaluating the impact of tailored implementation support on the uptake of MyPain/PainManager. Themes identified through this analysis will inform the development of targeted implementation strategies for each clinic and will be integrated with the quantitative results to specifically target clinics and organizational roles with lower baseline organizational readiness. In leveraging these findings to inform implementation, strategies specifically identified by participants at the individual clinic will be prioritized. These strategies will be supplemented with other relevant strategies from the literature based on CFIR constructs that align with the perceived readiness for each clinic.

Our study is not without limitations. While questions on support strategies in the interview guide were open-ended, due to time constraints, follow-up prompts were focused on strategies used in previous rollout efforts within the participating health system. This may limit the support strategies that were suggested by participants, who might have selected different strategies if they had been presented with all 73 ERIC strategies. Additionally, while data were collected across 8 clinics, they were limited to one health system and focused on adoption of one FHIR application, which limits the generalizability of our findings. Finally, while our sample size was sufficient to detect differences in ORIC scores by role, the smaller sample size at each individual clinic was a barrier for assessing clinic-level ORIC trends. Future studies could address these gaps by using mixed-methods to assess organizational readiness in different settings, with varied EHR-based tools, and by presenting participants with a broader range of potential strategies.

## Conclusions

This mixed methods analysis provides a holistic assessment of organizational readiness to implement a CDS tool for chronic non-cancer pain care across diverse primary care clinics in the University of Florida Health system. The findings from the ORIC survey revealed high overall levels of change efficacy and commitment among clinic providers and staff, with notable differences between roles and clinics. The qualitative interviews identified key barriers and facilitators to implementation, including workflow integration challenges and the need for support strategies tailored to individual clinics barriers. Overall, this study highlights the benefits of a mixed methods approach to assessing organizational readiness prior to implementation. The use of a rapid qualitative analysis approach in conjunction with the ORIC survey allowed for the timely extraction of actionable insights, which are crucial for the formative stages of CDS tool deployment. Future research should evaluate the impact of these tailored strategies on CDS tool adoption and patient outcomes.

## Data Availability

The raw data supporting the conclusions of this article will be made available by the authors, without undue reservation.
